# Artificial Intelligence for Organelle Segmentation in Live-Cell Imaging

**DOI:** 10.34133/research.1035

**Published:** 2025-12-16

**Authors:** Yang Ding, Zhijun Tan, Jintao Li, Weisen Zhang, Bin Fang, Hua Bai, Weini Xin, Nicolas H. Voelcker, Bo Peng, Lin Li

**Affiliations:** ^1^ State Key Laboratory of Flexible Electronics (LoFE) and Institute of Flexible Electronics (IFE), Northwestern Polytechnical University, Xi’an 710072, China.; ^2^ State Key Laboratory of Flexible Electronics (LoFE) and Institute of Flexible Electronics (IFE, Future Technologies), Xiamen University, Xiamen 361005, China.; ^3^ Hospital of Stomatology Shantou University Medical College, Shantou 515000, China.; ^4^ Department of Stomatology, Shantou University Medical College, Shantou 515000, China.; ^5^ Drug Delivery, Disposition and Dynamics, Monash Institute of Pharmaceutical Sciences, Monash University, Parkville, Victoria 3052, Australia.

## Abstract

Organelle morphology and dynamics are closely linked to cellular function and fate, yet their relationships remain poorly defined across physiological and pathological contexts. Live-cell imaging enables the visualization of subcellular structures and dynamic processes but often requires extensive manual analysis, introducing variability and limiting reproducibility and throughput. Image segmentation partitions digital images into meaningful regions, facilitating the quantification of organelle morphology and molecular behavior for precise subcellular analysis. Herein, this review surveys recent advances in live-cell imaging segmentation algorithms across diverse organelles, from traditional thresholding-based methods to deep learning approaches that enhance accuracy and adaptability in complex biological environments. We discuss key challenges, including 3-dimensional imaging, multi-organelle segmentation, and generalization across diverse imaging modalities. We also highlight label-efficient strategies, synthetic data, and physics-guided modeling that reduce reliance on manual annotations and large annotated datasets. By advancing generalist models, these innovations improve quantitative cell biology, accelerate disease research, and drive therapeutic discovery, underscoring the transformative role of artificial intelligence in biomedical microscopy.

## Introduction

**Fig. 1.**
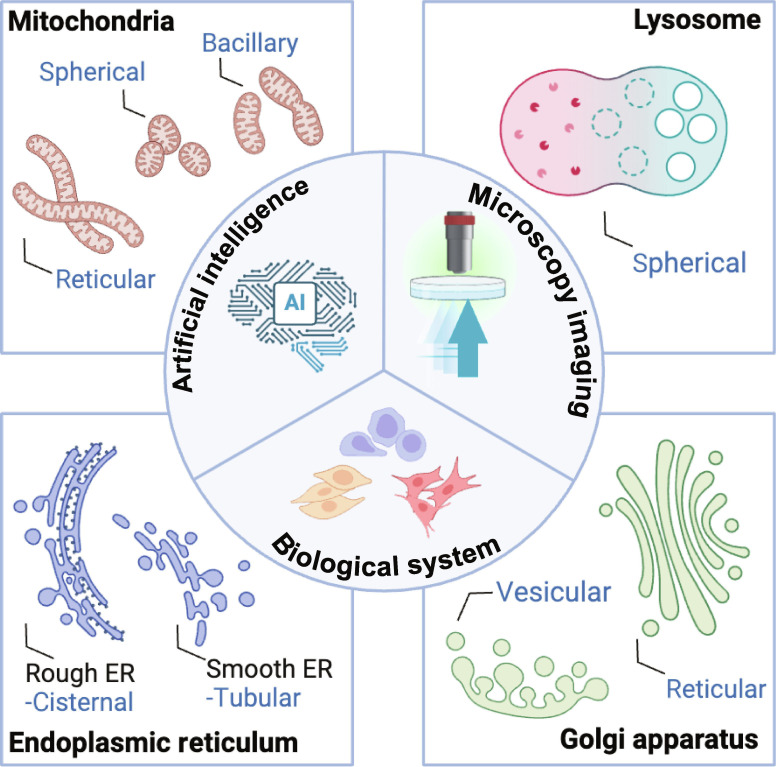
Artificial intelligence (AI)-integrated microscopy imaging for subcellular analysis. The integration of AI with microscopy imaging techniques facilitates detailed analysis of cellular organelles, including mitochondria, lysosomes, the ER, and the GA, thereby driving progress in biomedical research on subcellular systems (created with BioRender).

Cellular components are essential for executing complex biological processes that sustain organisms [1]. Detailed observation of organelle morphology is crucial for understanding cellular fate, as its dynamic architecture reflects functional states [2]. For instance, mitochondria generate energy and regulate programmed cell death through dynamic processes such as fission and fusion [3]. Similarly, the endoplasmic reticulum (ER) maintains cellular homeostasis by storing calcium and detoxifying substances, with its intricate topology underpinning multifunctionality [4]. Moreover, abnormalities in the morphology and dynamic of the Golgi apparatus (GA) and lysosomes are linked to neurodegenerative disorders, cancer, and metabolic diseases [5–7] (Fig. 1).

**Fig. 2.**
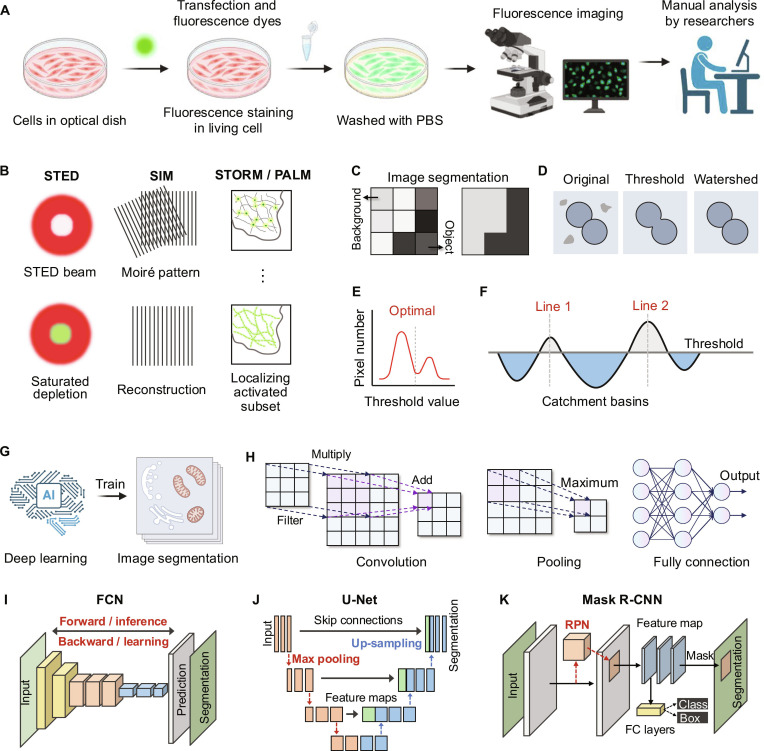
Protocols and flowchart depicting fluorescence imaging and image segmentation techniques. (A) The workflow for fluorescence imaging involves cell preparation, fluorescent staining, washing, and imaging, and then analyzed manually by researchers (created with BioRender). (B) SR fluorescence imaging techniques, such as STED (saturated depletion of fluorescence allows for the resolution of structures beyond the diffraction limit), SIM (Moiré patterns generated enhance resolution), and STORM/PALM (localization of activated fluorescent subsets), improve the visualization of subcellular structures with high spatial resolution. (C) Image segmentation achieves its results by separating the object from the background, creating a clear distinction between the two. (D) An example of the workflow for image segmentation methods. (E) Segmentation is achieved by selecting an optimal threshold value that separates pixel intensities into distinct categories. (F) Watershed algorithm segments an image by treating pixel intensities as topographic features. Catchment basins and watershed lines are identified, dividing the image into meaningful segments based on gradients. (G) Deep learning models are employed to train segmentation algorithms, enabling automated processing of subcellular fluorescence images (created with BioRender). (H) The principles of convolutional neural networks (CNNs) for image analysis are shown, including convolutional operations (multiplication with filters), pooling (down-sampling through maximum value extraction), and fully connected layers (integration of features for classification). (I) Fully convolutional network (FCN) employs forward inference and backward learning, transforming input images into segmented outputs [47]. (J) U-Net uses skip connections between the max pooling and the up-sampling, improving feature extraction and localization [48]. (K) Mask R-CNN architecture combining region proposal networks (RPN) with fully connected layers to predict masks, classes, and bounding boxes simultaneously for each object in an image [49].

Live-cell imaging is essential for examining cellular processes within their native environments [8–10]. Fluorescence imaging offers important advantages by enabling the selective visualization of target molecules through fluorescent labeling without requiring sample fixation, thus preserving cell viability [11,12]. This approach allows researchers to observe dynamic intracellular events in real time, providing more accurate insights into cellular functions and interactions [13] (Fig. 2A). However, conventional fluorescence microscopy is limited by the optical diffraction barrier (~200 nm), restricting the clear visualization of smaller subcellular structures [14]. Although scanning electron microscopy and transmission electron microscopy provide high-resolution imaging, their extensive sample preparation requirements, including fixation and dehydration, render them unsuitable for live-cell imaging and real-time tracking of intracellular biomolecules [15–17]. To address these constraints, super-resolution (SR) fluorescence imaging techniques have been developed [18,19]. Methods including stimulated emission depletion (STED) microscopy, structured illumination microscopy (SIM), and single-molecule localization techniques surpass the diffraction limit of light, enabling the real-time visualization of subcellular structures and processes at nanoscale resolution without compromising cell viability [20–23]. These advancements substantially enhance our understanding of cellular functions and disease mechanisms by facilitating the detailed examination of subcellular components and their interactions (Fig. 2B).

Despite advances in imaging techniques, analyzing the resulting image data presents challenges. Manual analysis is labor-intensive and impractical for large-scale studies, highlighting the need for automated image analysis methods [24,25]. Image segmentation, which divides an image into regions of interest (ROIs), is essential for quantifying structures and processes within microscopy images [26,27]. Methods such as thresholding and feature-based classifiers address these issues by leveraging handcrafted features such as texture, intensity gradients, and morphological descriptors. These approaches are computationally efficient and perform well in scenarios with clear target-background separation [28,29]. Moreover, deep learning-based methods excel at automatically extracting hierarchical features from raw images, enabling robust segmentation of diverse organelles even under low signal-to-noise ratios (SNRs). By applying appropriate segmentation approaches, researchers can achieve more efficient and accurate segmentation across various tasks, facilitating morphological quantification and advancing microscopy image analysis, thereby enabling deeper understanding of cellular functions and disease mechanisms [30,31].

In this review, we focus on the advancements in organelle image segmentation and analysis for live-cell imaging, emphasizing their transformative impact on biomedical research. We introduce the principles, methodologies, and applications of various segmentation techniques, highlighting their utility in studying subcellular structures. Moreover, we outlook future perspectives in this field, aiming to overcome current limitations and drive innovations in cell biology and pathology.

## Fundamentals of Image Segmentation

Image segmentation is a fundamental task in computer vision and image analysis that involves dividing images into meaningful areas corresponding to objects or ROIs [32]. It is critical for extracting quantitative information and forms the basis for various applications, including object recognition, tracking, and scene understanding [33] (Fig. 2C). In natural scenes, segmentation plays a pivotal role in identifying and delineating objects, supporting tasks such as autonomous driving, surveillance, and image retrieval [34]. Segmentation techniques are generally categorized into semantic and instance segmentation [35]. Semantic segmentation assigns a class label to each pixel, grouping regions based on shared characteristics but without distinguishing between individual instances of the same class [36]. On the other hand, instance segmentation not only classifies pixels but also identifies individual instances within a class, providing detailed insights into object boundaries and spatial relationships [37]. This approach is particularly valuable in biomedical imaging, where precise delineation of structural details is essential for analysis and interpretation [38].

Thresholding and the watershed algorithm are widely used in image segmentation [39,40] (Fig. 2D). Thresholding categorizes pixels into foreground and background regions based on intensity values, offering computational efficiency in many scenarios [41] (Fig. 2E). The watershed algorithm treats the image as a topographical surface and segments regions based on intensity gradients, making it effective for separating touching or overlapping objects (Fig. 2F). In practice, the watershed is often complemented by postprocessing steps, such as entity separation and marker-based adjustments, to optimize accuracy and performance [42]. Although these methods have been successfully applied to both natural and microscopy image analysis, challenges such as the heterogeneity of biological structures, elevated noise levels, and variations in signals require careful consideration during complicated segmentation tasks [43].

Deep learning has revolutionized image segmentation by enabling models to learn hierarchical feature representations directly from data [44] (Fig. 2G). Convolutional neural networks (CNNs), a fundamental deep learning architecture, play a crucial role in segmentation by automatically identifying and distinguishing patterns across different feature levels [45,46] (Fig. 2H). Additionally, advanced architectures, such as fully convolutional networks (FCNs), U-Net, and mask region-CNN (Mask R-CNN), have demonstrated robustness to variations in lighting, occlusion, and object appearance [47–49]. FCNs, designed for semantic segmentation, replace fully connected layers with convolutional layers to generate pixel-wise predictions [47] (Fig. 2I). U-Net, with its symmetric U-shaped architecture, captures both local and global context, making it particularly effective for biomedical image segmentation, even with limited training data [48] (Fig. 2J). Mask R-CNN extends object detection models by predicting object masks alongside bounding boxes, enabling precise instance segmentation [49] (Fig. 2K). These deep learning algorithms have gained widespread adoption in microscopy imaging. By enabling precise segmentation of subcellular structures, it supports accurate morphological quantification and dynamic tracking. These improvements contribute to a deeper understanding of biological processes at subcellular levels [50,51].

The evolution from traditional to deep learning-based segmentation represents a fundamental paradigm shift in computational image analysis. Classical methods remain interpretable and computationally efficient for high-contrast, well-defined structures but often require extensive parameter tuning and tend to fail when applied to heterogeneous or dynamic organelle morphologies [52]. Deep learning addresses these limitations through end-to-end feature learning, allowing models to automatically extract hierarchical representations directly from data [53]. This data-driven approach enables robust segmentation of complex structures such as mitochondrial networks or ER tubules under varying SNRs and imaging modalities, providing superior accuracy and generalization that manual feature design cannot match. In practical applications, users typically select segmentation models based on the characteristics of their specific tasks (Table 1).

**Table 1.** Summary of common segmentation approaches for live-cell organelle imaging

Algorithm type	Method	Advantages	Typical scenarios	Application limitations
Traditional	Thresholding	Fast, interpretable, and minimal setup	Quick analysis of bright, isolated organelles or initial process	Requires careful parameter tuning for each dataset and struggles to handle touching objects
	Watershed algorithm	Effective at separating touching objects	Segmenting moderately separated objects after preprocessing	Sensitive to parameters and unsuitable for thin tubules or highly fused networks
Deep learning-based	CNNs	Extracts local features and achieves efficiency through parameter sharing	Small, consistent organelles with repeatable texture	Suitable for initial CNN prototypes but requires many labeled patches and careful border handling
	FCN	Provides dense, end-to-end prediction over entire images	Semantic segmentation of homogeneous organelle populations	Struggles to capture fine details; often requires boundary refinement for precise segmentation
	U-Net	Multiscale feature fusion preserves fine detail and contextual information	Widely effective for 2D/2.5D organelle segmentation	Extendable to 3D but computationally demanding; sensitive to anisotropy
	Mask R-CNN	Produces accurate instance masks and separates touching objects	Instance-level tasks such as cell counting or single-organelle analysis	Requires instance separation; high annotation cost and tuning for small or tubular structures

Accurate evaluation of image segmentation is crucial for developing reliable analysis tools in microscopy. Segmentation quality is typically assessed by comparing predictions with ground truth (GT) annotations using quantitative metrics [54] (Table 2). Commonly used metrics include pixel-level, region-based, and boundary-based measures, each providing distinct insights into performance. Pixel-level metrics, such as accuracy, evaluate individual pixel classification and are suitable for simple, high-contrast tasks or class-wise error analysis. On the other hand, object-level scores such as precision/recall and average precision, evaluate instance detection, counting, and separation. For networked or 3-dimensional (3D) organelles, topology/connectivity measures and axis-aware error statistics (e.g., skeleton overlap, topology preservation, and *z*-axis localization errors) better capture biologically relevant structure [55]. Region-based metrics, for example, the Dice coefficient and Jaccard index (Intersection over Union), measure the overlap between predicted and GT regions, preferred for assessing volumetric/area agreement on complex morphologies [56]. These metrics are particularly effective for datasets with complex morphologies. Boundary-based metrics, including the Hausdorff distance, evaluate the spatial accuracy of object contours, focusing on the maximum discrepancy between predicted and GT boundaries. This is especially critical in high-resolution microscopy, where precise boundary localization is essential for downstream analyses. By combining these metrics, researchers can comprehensively evaluate segmentation algorithms, identify weaknesses, and iteratively refine methods to optimize performance for challenging microscopy datasets.

**Table 2.** Summary of confusion matrix components and performance metrics used for evaluating image segmentation models

Confusion matrix	Definition	
True positive (TP)	Correctly predicted positive pixels	
False positive (FP)	Incorrectly predicted positive pixels (actual negative)	
True negative (TN)	Correctly predicted negative pixels	
False negative (FN)	Incorrectly predicted negative pixels (actual positive)	
Performance metrics	Definition	Calculation formula
Pixel accuracy (PA)	Fraction of correctly classified pixels over all pixels	(TP + TN) / (TP + TN + FP + FN)
Mean pixel accuracy (MPA)	Average of pixel accuracy across all classes	[TP / (TP + FP) + TN / (TN + FN)] / 2
Recall	Fraction of true positives among actual positives	TP / (TP + FN)
Precision	Fraction of true positives among predicted positives	TP / (TP + FP)
Specificity	Fraction of true negatives among actual negatives	TN / (TN + FP)
Dice coefficient	Measure of overlap between predicted and GT regions	2 × TP / (2 × TP + FP + FN)
Intersection over union (IoU)	Ratio of the intersection area to the union area between prediction and GT	TP / (TP + FP + FN)
Mean intersection over union (mIoU)	Average IoU across all classes	[TP / (TP + FP + FN) + TN / (TN + FN + FP)] / 2

## Organelle Image Segmentation

Investigations into organelles illuminate the intricate interplay of cellular systems, uncovering how specialized structures orchestrate homeostasis, regulate metabolic pathways, and modulate signal transduction. The structural and functional integrity of organelles, including mitochondria, ER, GA, and lysosomes, is critical for cellular health. Deviations in organelle shape and behavior are frequently associated with disease development [51]. Consequently, precise characterization of organelles is crucial for advancing our understanding of cell biology and mechanisms.

Organelle image segmentation is important for extracting precise spatial and structural information, forming the foundation for subsequent quantitative analyses. Unlike whole-cell or nuclear, organelle segmentation is inherently more challenging due to the smaller size, irregular shapes, and intricate distributions of these structures. Additionally, many organelles exhibit dynamic behaviors such as fusion, fission, and trafficking, requiring accurate segmentation across both temporal and spatial dimensions. Advances in segmentation technologies have notably improved the ability to identify and characterize organelles with high-precision accuracy, opening new avenues for understanding cellular functions in health and disease.

### Mitochondrial image segmentation

Mitochondria, referred to as the cell’s “powerhouses”, are central to energy production and play key roles in a range of essential biochemical reactions. Their dynamic behavior, characterized by fission, fusion, and structural changes, reflects cellular states and stress responses [57] (Table 3).

**Table 3.** Summary of representative algorithms and techniques for mitochondrial image segmentation and analysis

Algorithm	Technique	Applications	Modality	Performance	3D capability
CellProfiler [58]	Otsu’s and adaptive thresholding and random forest	Segmentation and classifies various morphology in MCF-7 cells	Wide-field deconvolution microscopy	–	No
MitoGraph [59]	Graph-theory-based image analysis tool	2D/3D network analysis in various cell types	Confocal microscopy	Voxel-level accuracy > 0.90	Yes
Mitochondria Analyzer [60]	Subtract background and adaptive thresholding	2D/3D morphology feature analysis in MIN6 and pancreatic β-cells	Laser scanning confocal microscopy	–	Yes
MiNA [61]	IsoData thresholding	Network features analysis in SH-SY5Y cells, C2C12 cells, and MEFs cells	Fluorescence microscopy	–	No
MitoSPT [62]	Fast Fourier transform and manually thresholding	Motion analysis in cells under the impact of cytoskeletal inhibitors and metabolic interactions	Fluorescence microscopy	–	No
Mitometer [65]	Gaussian filter, intensity threshold, and global assignment tracking algorithm	2D/3D dynamics analysis in normal breast epithelial, receptor-positive breast cancer, and triple-negative breast cancer cells. Studying motility, morphology, and their correlation with metabolic activity	Laser scanning confocal microscopy	–	Yes
MitoSegNet [66]	U-Net	Morphology quantitative in *C. elegans* cells, Catp-6ATP13A2 Mutants *vs.* Wild-type, HeLa cells	Fluorescence microscopy	Dice = 0.89	No
Sekh et al. [67]	Simulation-supervised learning, EfficientNet-B3, U-Net	Segmentation and analysis including morphological and temporal event tracking	Structured illumination microscopy	mIoU = 0.74–0.76, F1 = 0.79–0.84	No
Somani et al. [68]	GAN, DNN, U-Net	Segmentation and tracking of dynamics in H9c2 cells	Fluorescence microscopy	mIoU = 0.78	No
MoDL [69]	U-Net, ResNets, convolutional block attention modules	Segmentation and morphology feature analysis in various cell types and imaging modalities	Structured illumination microscopy	Dice = 0.92, mIoU = 0.84, PA = 0.95	No

The segmentation of mitochondrial images is critical for quantitative analyses of their morphology, dynamics, and network structures. In 2012, Reis et al. [58] utilized CellProfiler to segment mitochondria and classify their morphologies into networked, fragmented, and swollen categories using a Random Forest algorithm. In 2015, Viana et al. [59] proposed the MitoGraph, a tool for 3D mitochondrial analysis that converts mitochondrial networks into skeletonized representations to quantify volume and length. Complementing these advancements, Mitochondria Analyzer [60] and MiNA [61], the ImageJ plugins, were developed to provide user-friendly tools for detailed analysis of mitochondrial networks, extracting skeletonized parameters such as branch length and connectivity. Additionally, the integration of dynamic tracking algorithms, such as MitoSPT [62], and image restoration tools, including CARE [63] and Noise2Void [64], has further enhanced segmentation accuracy, enabling analysis of mitochondrial dynamics and morphology across varying imaging conditions.

**Fig. 3.**
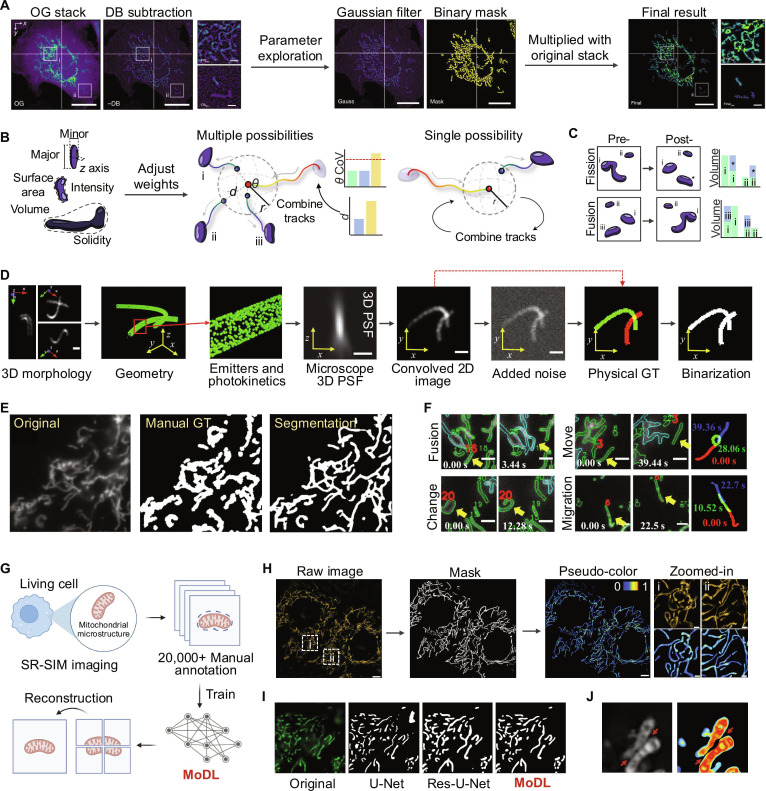
Mitochondrial image segmentation and analysis in living cells. (A) The Mitometer segmentation pipeline processes the original (OG) image through a diffuse background (DB) subtraction algorithm to reduce noise between adjacent mitochondria. Gaussian filtering and area thresholding are then applied to generate a binary mask, which is multiplied with the OG stack to produce the output. (B) The Mitometer tracking pipeline evaluates confident mitochondrial tracks using an interCoV:intraCoV ratio for each morphological parameter. Gap-closing is resolved by assessing travel angle (*θ*) and center-of-mass distance (*d*), while single possible track combinations are merged automatically. (C) Fission and fusion events are detected by Mitometer through analyzing changes in volume and extrema distances of nearby mitochondria before and after the events [65]. (D) The simulation pipeline includes generating 3D mitochondrial geometry and photokinetics of fluorescent molecules. A microscope’s 3D PSF is applied to generate synthetic 2D images. Noise is added using a microscopy noise model to create realistic images, and segmentation masks are computed from the noise-free images. (E) Manual segmentation demonstrates variability influenced by annotators’ knowledge, making it unsuitable for supervised learning approaches due to inconsistencies. (F) Examples of mitochondrial fusion, fission, movement, flipping, and migration are captured using segmentation and tracking, showcasing the utility of the proposed method for identifying dynamic events in living cells [67]. (G) Schematic illustrating the key steps of MoDL, a deep learning-based method for precise mitochondrial segmentation. (H) MoDL segmentation generates binary masks from original images, which are then multiplied by fluorescence intensity to produce pseudo-color images reflecting individual mitochondrial fluorescence. (I) Enhancement of the segmentation capability in detail through the improvements made in U-Net, Res-U-Net, and MoDL. (J) MoDL accurately distinguished 2 closely attached mitochondria [69]. Reprinted from Refs. [65,67,69] with permission from Springer Nature.

Mitometer, introduced by Lefebvre et al. in 2021 [65], represents an advancement in the segmentation and tracking of mitochondria, addressing challenges in analyzing their morphology and dynamic behavior in live-cell fluorescence time-lapse images. This automated tool combines size- and shape-preserving background removal with Gaussian smoothing and intensity thresholding to generate stable segmentation masks. Its robust algorithm isolates individual mitochondria, even in crowded perinuclear regions, ensuring accurate segmentation in both 2-dimensional (2D) and 3D datasets (Fig. 3A). For dynamic tracking, Mitometer employs a frame-by-frame global optimization approach based on morphological and spatial displacement parameters, enabling the identification of mitochondrial motion, fission, and fusion events (Fig. 3B and C). Moreover, the tool revealed significant differences in mitochondrial motility and morphology between triple-negative breast cancer, estrogen receptor-positive, and normal epithelial cells. By integrating NADH fluorescence lifetime imaging, Mitometer established correlations between mitochondrial dynamics and metabolic states, providing insights into the role of mitochondria in cancer aggressiveness. With its user-friendly interface, Mitometer becomes a versatile tool for advancing mitochondrial research and clinical applications.

However, the inherent complexity of mitochondrial morphology, including fragmented, networked, or overlapping structures, pose substantial challenges for thresholding algorithms, particularly under diverse imaging conditions or in datasets with varying SNRs. Fischer et al. [66] introduced MitoSegNet, a deep learning-based segmentation framework specifically designed for mitochondrial morphology analysis. Built on a modified U-Net architecture, MitoSegNet was trained using fluorescence microscopy images of *Caenorhabditis elegans*, enabling it to adapt to diverse mitochondrial morphologies, including elongation, fragmentation, and tubular. Despite its use of deep learning techniques, MitoSegNet lacks a thorough exploration of algorithmic applicability and potential applications. While effective for morphological segmentation, it does not adequately address challenges such as the variability of mitochondrial structures across different cell types and imaging conditions.

Deep learning has advanced mitochondrial segmentation by automating feature extraction, yet its reliance on extensive high-quality datasets remains an important bottleneck. Moreover, the complex structures of mitochondria and the limitation of optical microscopy render accurate GT labeling labor intensity and inconsistent. To address these challenges, Sekh et al. [67] proposed a simulation-supervised approach that generates realistic simulated images of mitochondria. This method incorporates physical models of microscopy, including the 3D point spread function (PSF), fluorescence labeling, and noise characteristics, to create training data (Fig. 3D). By training U-Net-based models on this simulated dataset, the method achieved superior segmentation results on live-cell imaging and enabled detection of mitochondrial dynamic events (Fig. 3E and F). Similarly, Somani et al. [68] introduced a physics-guided virtual labeling framework that employs a conditional deep neural network with a PSF-based loss function to convert bright-field into fluorescence-like images. It prioritizes critical foreground features while deprioritizing noisy backgrounds, bridging the gap between fluorescence-based and label-free imaging. These methods highlight the potential of combining physics-based modeling with deep learning to enhance mitochondrial segmentation under specific imaging conditions.

We recently introduced MoDL [69], a deep learning framework for high-precision mitochondrial segmentation. Trained on an extensive dataset of SR fluorescence images, MoDL demonstrated robust performance across diverse imaging platforms and heterogeneous cell types (Fig. 3G and H). Based on U-Net architectures, MoDL integrates residual networks and convolutional block attention modules, enabling the extraction of deeper image features and enhancing learning robustness by addressing issues such as gradient vanishing and exploding (Fig. 3I). MoDL excels in capturing intricate mitochondrial details, including resolving closely connected structures, and outperforms previous models in segmentation accuracy (Fig. 3J). Notably, it incorporates an ensemble learning strategy and conducts detailed analysis of complex mitochondrial morphological parameters, enabling accurate predictions of diverse mitochondrial functions. This framework extends its applications to high-throughput morphological analysis, functional abnormality screening, and the study of the intricate interplay between mitochondrial structure and function in various biological contexts.

Despite advancements in mitochondrial image segmentation techniques, substantial challenges persist, such as accurately distinguishing overlapping and morphologically diverse structures, managing low SNRs, and addressing dynamic behaviors like fission and fusion. The absence of standardized, annotated datasets further limits model training and reduce generalizability across imaging modalities. Future advancements are expected to integrate self-supervised and physics-informed learning to minimize reliance on manual annotations, thereby enhancing training efficiency. Moreover, multimodal frameworks tailored for diverse imaging conditions could improve adaptability and robustness. Progress in spatiotemporal tracking techniques will further enable comprehensive analysis of mitochondrial dynamics in live-cell imaging, paving the way for more advanced insights into cellular processes.

### ER image segmentation

The ER is a highly dynamic organelle essential for cellular homeostasis, playing diverse and indispensable roles in various cellular functions. The ER consists of 2 distinct regions: the rough and smooth ER. Together, these regions form a continuous, complex network that extends throughout the cytoplasm, enabling intracellular communication and interactions with other organelles. The ER’s topology reflects its functional diversity, comprising an intricate network of interconnected sheets, tubular structures, and cisternae [4] (Table 4).

**Table 4.** Summary of representative algorithms and techniques for ER image segmentation and analysis

Algorithm	Technique	Applications	Modality	Performance	3D capability
VGG-UNet/VGG-SegNet [71]	VGG19, U-Net, SegNet	Tested on the FMI dataset with segmentation accuracy >98%	Spinning disk confocal microscopy	VGG-UNet: JA = 0.97, Dice = 0.98, ACC = 0.99; VGG-SegNet: JA = 0.96, Dice = 0.98, ACC = 0.99	No
AnalyzER [75]	Graph-theoretic metrics, optical flow, phase congruency enhancement	Segmentation and dynamic analysis of plant networks in nicotiana tabacum leaf epidermal cells and *Arabidopsis*	Confocal microscopy	Dice = 0.94 ± 0.019	No
ERnet [77]	Vision-Transformer and multihead self-attention	Segmentation and topological analysis of ER networks on COS-7, HEK293T, CHO, and U2OS cells. Applicable to model of hereditary spastic paraplegia, Niemann–Pick disease, and metabolic stress.	Structured illumination, wide-field, and confocal microscopy	Segmentation: PA = 0.92–0.99; node identification: ACC = 0.96–0.99; robust to noise: SNR >5	Yes

**Fig. 4.**
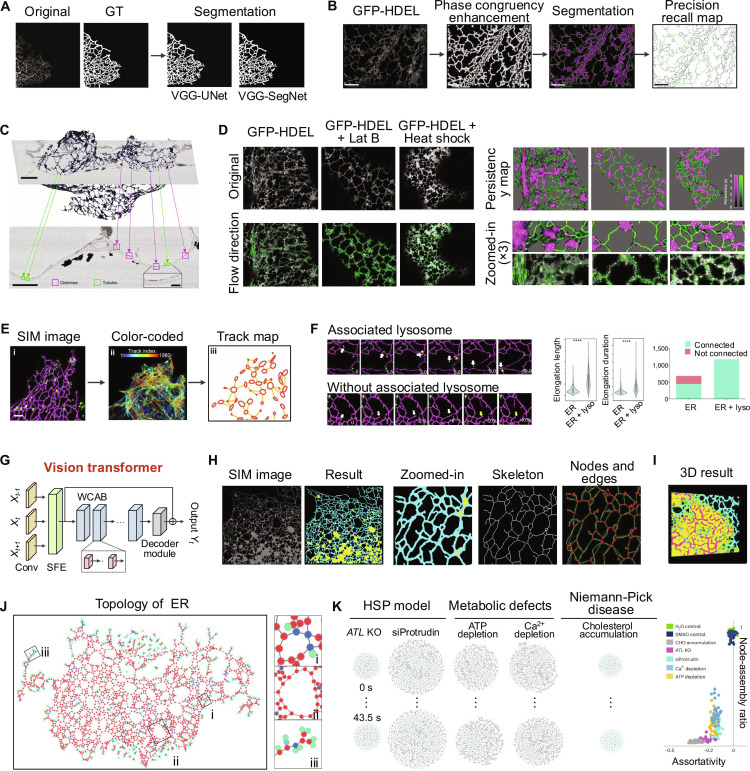
ER image segmentation and analysis in living cells. (A) ER segmentation results are generated using 2 deep learning architectures, VGG-UNet and VGG-SegNet [71]. (B) AnalyzER processes a paradermal optical section of a tobacco leaf epidermal cell expressing GFP-HDEL, an ER lumenal marker. Tubular elements are enhanced using phase congruency via the local weighted mean phase angle, followed by hysteresis thresholding and thinning to generate a single-pixel skeleton. Cisternal structures are segmented using active contour refinement, and the skeleton is validated against manually digitized GT. (C) AnalyzER generates a 3D reconstruction of the ER, with enlarged cross-sections highlighting tubular ER elements and detailed insets of specific regions. (D) Using AnalyzER, ER network dynamics and persistency are mapped. Treatments with Lat B or heat shock reveal effects on network movement. Persistency maps, calculated with a 12-frame lag, highlight cisternae, tubules, and nodes, with enlarged regions visualizing central areas and arrows indicating predicted movement directions [75]. (E) Lu et al. utilized SIM to image the ER and lysosomes in COS-7 cells. A track density map was generated from single-particle tracking (SPT) data. (F) This work revealed that newly formed ER tubules exhibit coupled motion with lysosomes. Quantitative analysis highlights differences in ER dynamics with and without lysosome association [76]. (G) ERnet employs a vision transformer architecture with a shallow feature-extraction module that projects input images into feature maps. Residual window channel attention blocks (WCAB), containing Swin Transformer layers (STLs), enhance feature extraction and segmentation. (H) ERnet segmentation of video-rate SIM image reveals detailed ER structures. Zoomed-in regions distinguish closely positioned nodes, while skeletonized networks illustrate nodes and edges within the ER. (I) Using ERnet, a volumetric 3D reconstruction from SIM sectioning reveals sheet-based tubules (SBTs) embedded within sheet domains. (J) ERnet represents the ER tubular network topology as a connectivity graph, highlighting multiway junctions, polygonal structures formed by 3-way junctions and tubules, and growth tips of ER tubules. (K) ERnet quantitatively analyzes ER phenotypes in disease models, including hereditary spastic paraplegia (HSP), metabolic defects, and Niemann–Pick disease. Metrics like node centrality and assortativity reveal structural and functional ER alterations [77]. Reprinted from Ref. [71] with permission from Wiley; from Ref. [75,77] with permission from Springer Nature; and from Ref. [76] with permission from the American Association for the Advancement of Science.

Accurate segmentation of ER images is critical for understanding its morphology, spatial organization, and dynamic interactions with other organelles. The thin, branched, and interconnected architecture of the ER presents important challenges for image analysis. Advanced segmentation methods that resolve the ER’s fine-grained details are essential for extracting biologically meaningful data, enabling the study of ER-associated processes in cellular health and disease [70]. To address these challenges, Daniel et al. [71] developed a CNN-based automated segmentation framework for analyzing ER networks in fluorescence microscopy images. This study introduced VGG-UNet and VGG-SegNet, enhanced versions of the U-Net and SegNet architectures that incorporated the VGG19 backbone for improved feature extraction and segmentation accuracy. Comparative evaluations revealed that VGG-SegNet consistently outperformed other methods, including U-Net [72], SegNet [73], and Res-UNet [74] (Fig. 4A). This advancement indicates the potential of VGG-SegNet as a robust tool for precise ER segmentation in fluorescence microscopy research. However, while these methods improved segmentation accuracy, they primarily emphasized performance metrics and provided limited insights into the morphology and dynamic properties of ER networks. Addressing these gaps will require future approaches that integrate segmentation with tools for analyzing the functional and dynamic characteristics of ER’s topological structure.

Expanding on previous advancements, Pain et al. [75] introduced AnalyzER, a robust and quantitative framework for analyzing the dynamics and morphology of the plant ER. AnalyzER integrates fluorescence and electron microscopy data to automate the segmentation of ER substructures. An intensity-independent phase congruency filter enhances tubular features across scales and orientations. Subsequently, tubules are segmented using hysteresis thresholding and skeletonized via a thinning algorithm, while cisternae are isolated through morphological opening followed by active contouring for boundary refinement. Graph-theoretic metrics are then used to quantify network topology and facilitate extraction of enclosed polygonal regions (Fig. 4B and C). Additionally, the platform incorporates persistency mapping to identify static ER elements and employs optical flow algorithms to quantify the velocity and directionality of ER movement. AnalyzER has demonstrated its versatility by quantitatively capturing changes in ER morphology and dynamics under various pharmacological and environmental perturbations, such as Latrunculin B, Brefeldin A, and heat shock (Fig. 4D). These capabilities make AnalyzER a comprehensive tool for studying protein-specific roles in ER morphology and the dynamic interplay between ER structure and cellular physiology.

In 2020, Lu et al. [76] advanced the understanding of ER topology by investigating its dynamic remodeling mechanisms and the relationship between ER and lysosomal motion. This study utilized SR fluorescence imaging to visualize and quantify the dynamic interactions between ER and lysosome tubules. For quantitative analysis, thresholding techniques were employed to identify ROIs within fluorescence images, effectively distinguishing ER structures from background noise (Fig. 4E and F). Building on this foundation, the same group introduced ERnet in 2023, a deep learning-based tool for semantic segmentation and quantitative analysis of ER topology [77]. ERnet leverages a Vision Transformer architecture, incorporating Swin Transformer layers with multihead self-attention (MSA) and channel attention mechanisms to process spatiotemporal image data (Fig. 4G). The model effectively segments ER domains into tubular and sheet structures, followed by skeletonization and representation using graph theory (Fig. 4H). Additionally, ERnet supports 3D segmentation by processing temporal image sequences as 3D blocks, enabling robust analysis of dynamic ER networks (Fig. 4I). Furthermore, ERnet models the complex topology of ER tubular networks represented by a connectivity graph, allowing for detailed visualization and quantification of structural elements (Fig. 4J). Under genetic and metabolic perturbations, ERnet quantitatively captures phenotypic changes, such as increased network fragmentation and altered connectivity (Fig. 4K). These capabilities position ERnet as a powerful tool for studying ER dynamics and disease-associated phenotypes with high precision and reproducibility.

ER image segmentation presents unique challenges, including the intricate topology of tubular and cisternal regions and the rapid transitions between structural states. The variability in fluorescence intensity along ER branches, combined with fragmented or poorly labeled regions, further complicate the segmentation process. Future advancement could focus on adaptive models capable of dynamic feature extraction. Moreover, incorporating topological analysis directly into segmentation frameworks may enable more accurate characterization of complex ER networks.

### Nuclei image segmentation

The nucleus is the largest and most frequently segmented subcellular structure, serving as a primary indicator for cell state, cell cycle, ploidy, and pathological changes. Accurate nuclear masks enable measurements of nuclear size, shape, chromatin condensation, mitotic figures, and spatial relationships to other organelles, which are all essential parameters in quantitative cell biology and pathology. Although global thresholding and watershed methods remain useful for isolated, high-contrast nuclei, common challenges include clumped or overlapping structures, mitotic morphology, and variable staining intensity across samples [78]. Consequently, modern approaches integrate instance-aware models, label-free, and 3D-aware segmentation for z-stacks (Table 5).

**Table 5.** Summary of representative algorithms and techniques for nuclei image segmentation and analysis

Algorithm	Technique	Applications	Modality	Performance	3D capability
Mayala and Haugsøen [79]	Multi-threshold averaging, Otsu binarization, and marker-controlled watershed	Nucleus, cytoplasm, and cell body extraction in white blood cell	Brightfield microscopy	JI = 0.903, Specificity = 0.993, Sensitivity = 0.953, Precision = 0.949	No
Mustafa et al. [80]	Gamma correction and multi-Otsu thresholding	Automated cervical nuclei segmentation in Pap smear cytology for cancer screening	Brightfield microscopy (Pap smear images, Herlev dataset)	F1 = 0.97, Precision = 0.99, Sensitivity = 0.95	No
Sumon et al. [81]	Comparison of random forest, support vector machine (SVM), k-nearest neighbor (kNN), naïve Bayes	Nuclei segmentation and classification in histopathological tissue images for cancer diagnosis and morphological analysis	Brightfield microscopy	Random forest: Dice = 0.96, IoU = 0.92; SVM: Dice = 0.95, IoU = 0.91; kNN: Dice = 0.93, IoU = 0.87; Naïve Bayes: Dice = 0.92, IoU = 0.85	No
CellSeg [82]	Pre-trained Mask R-CNN, mask expansion, and lateral bleed compensation	Segmentation and single-cell quantification in highly multiplexed fluorescence images (e.g., colorectal cancer CODEX datasets)	Multiplexed fluorescence microscopy	Dice = 0.9176, Recall = 0.9222	No
NISNet3D [84]	3D U-Net backbone and spatial attention modules	3D nucleus instance segmentation across multiple cell types	Fluorescence microscopy	PA = 0.94, Recall = 0.91, F1 = 0.93	Yes
Asmar et al. [85]	Physics-informed U-Net	Label-free nucleus segmentation and biophysical parameter quantification (phase retrieval, nuclear boundary detection)	Phase contrast and fluorescence microscopy	2D: F1 = 0.92–0.95; 3D: F1 = 0.70–0.78	Yes
Sistermanns et al. [86]	Automatic thresholding and plausibility checks	Cell and nucleus segmentation in unstained cervical cytology samples for diagnostic screening	Off-axis digital holographic microscopy	–	No
Luna et al. [87]	SAM, domain alignment module, federated style loss and multiclass hinge loss	Automatic nuclei segmentation and classification (frequent and rare nuclei types) in H&E histopathology	H&E histopathology images	Validation set: F1 = 0.733; Test set: F1 = 0.639	No

In 2022, Mayala and Haugsøen [79] proposed an automatic thresholding method based on local minima of image histograms, effectively segmenting nuclei and cytoplasm using B-spline approximation and multi-thresholding. Mustafa et al. [80] further advanced this direction by combining adaptive gamma correction with multi-Otsu thresholding to achieve high segmentation accuracy without the need for extensive annotated datasets. Although effective on benchmark datasets such as Herlev, these classical approaches remain limited in robustness and scalability across diverse imaging conditions. Recognizing these constraints, Sumon et al. [81] systematically compared a range of machine learning approaches and demonstrated that deep learning models consistently outperformed traditional techniques in both accuracy and generalization.

Building on these advances, deep learning has increasingly enabled biologically meaningful analyses in complex environments. In 2022, Lee et al. [82] developed CellSeg, an automated pipeline for nucleus segmentation in multiplexed tissue images that facilitated key discoveries in spatial biology. Built on a pretrained Mask R-CNN with mask expansion and lateral bleed compensation, CellSeg produced high-quality single-cell masks and allowed precise quantification of spatially organized “cellular neighborhoods” in colorectal cancer, revealing their role in antitumor immunity. To further enhance model adaptability and iterative refinement, Tomar et al. [83] introduced FANet, which incorporates a feedback attention mechanism that propagates the previous epoch’s prediction mask forward as a hard attention guide through a MixPool block, progressively improving segmentation accuracy. Evaluated on the 2018 Data Science Bowl dataset, FANet achieved state-of-the-art performance, illustrating the growing sophistication of deep learning models in nucleus segmentation.

As imaging technologies evolve toward volumetric and label-free modalities, new segmentation frameworks have emerged to tackle the challenges of 3D data. In 2023, Wu et al. [84] addressed the bottleneck in 3D tissue cytometry by developing NISNet3D, a deep learning-based method for nuclei instance segmentation in fluorescence microscopy. It uses a modified 3D U-Net, watershed transform, and synthetic data generation to reduce reliance on manual annotations. In 2024, Asmar et al. [85] developed a label-free artificial intelligence (AI) pipeline using 2D and 3D U-Nets to segment and track individual induced pluripotent stem cell nuclei and mitotic events from phase contrast images, leveraging automated fluorescence-based training. This enables noninvasive, high-accuracy quantification of single-cell dynamics over long periods, with potential for broader applications in stem cell monitoring and manufacturing.

Emerging strategies and foundational models have also been increasingly applied to nucleus segmentation in recent years. In 2024, Sistermanns et al. [86] developed an unsupervised multistage segmentation method for quantitative phase images, enabling efficient nucleus detection without manual annotation. This method achieved high throughput and low false-negative rates, and addressed the bottleneck in automated cervical cancer screening, with potential for clinical diagnostics and extended feature analysis in cytopathology. To address the challenge of long-tailed nuclei distribution in histopathology, in the same year, Luna et al. [87] enhances the Segment Anything Model (SAM) with category descriptors and domain alignment for automatic nuclei segmentation and classification. The model greatly improves detection of rare nuclei types while maintaining compatibility with manual prompts. It generalizes well to the Lizard dataset and offers promising extensibility to other medical imaging domains, with further optimization anticipated to reduce computational demands.

### Other organelles’ image segmentation

The cellular landscape includes numerous additional organelles that collectively sustain homeostasis through coordinated metabolic activities. The GA is involved in posttranslational modification, sorting, and trafficking of proteins and lipids, which are vital for cellular secretion and membrane integrity [5]. Lysosomes serve as the cell’s degradation centers, recycling macromolecules and removing dysfunctional organelles [6]. Meanwhile, lipid droplets (LDs) act as dynamic reservoirs for neutral-lipid, coordinating energy balance and form extensive contact sites with multiple organelles [88]. Peroxisomes contribute to fatty-acid β-oxidation, reactive oxygen species (ROS) metabolism, and lipid turnover, and their abundance and morphology dynamically respond to the cellular metabolic state [89]. Accurate segmentation of these organelles is crucial for studying their morphology, dynamics, and functional pathways. Advanced segmentation approaches enable quantitative analyses and high-throughput screening, providing deeper insights into cellular processes and pathological mechanisms (Table 6).

**Table 6.** Summary of representative algorithms and techniques for other organelles image segmentation and analysis

Algorithm	Technique	Applications	Modality	Performance	3D capability
**Golgi apparatus (GA)**					
2D-GolgiTrack [90]	Adaptive normalization thresholding, k-nearest neighbor matching	Quantitative analysis of dynamics, including fission, fusion, and morphological evolution in HeLa cells	Phase contrast and fluorescence microscopy	–	No
Sansaria et al. [91]	Otsu’s thresholding, watershed, logistic regression	Quantitative analysis and classification of dispersed versus undispersed Golgi images in HeLa cells.	Off-axis digital holographic microscopy	–	No
**Lysosomes**					
LysoQuant [92]	Enhanced U-Net version	Segmentation and analysis of lysosome-driven pathways, including recov-ER-phagy, ERLAD, and ER-phagy, in MEF and HEK293 cells	Confocal microscopy	Empty EL: IoU = 0.881 ± 0.012, F1 = 0.752 ± 0.134; Loaded EL: IoU = 0.877 ± 0.014, F1 = 0.814 ± 0.100	No
**Lipid droplets (LDs)**					
Dejgaard and Presley [93]	Adaptive thresholding and watershed-based separation	Quantitative analysis of LDs number, size, and distribution in mammalian hepatocytes	Confocal microscopy	–	No
Li et al. [94]	Improved watershed algorithm with distance transform and contour filtering	Segmentation and classification of LDs in liver pathology images (steatosis vs. nonsteatosis)	Brightfield microscopy	–	No
Sheneman et al. [95]	U-Net, random forest, XGBoost	Quantitative measurement of LDs number, size, and area fraction in 3T3-L1 and HeLa cells	Quantitative phase imaging system	Dice = 0.8668, Precision = 0.8778, Recall = 0.8691	No
Oh et al. [96]	U-net and HRNet	Detection of cells and intracellular LDs to predict lipid content during bio-fermentation	Confocal microscopy	Cell detection accuracy: IoU > 0.98; LDs detection accuracy: IoU > 0.92	No
**Peroxisomes**					
Perox-per-cell [97]	YeastSpotter CNN and Allen Cell Segmenter (LoG filtering)	Automated quantification of peroxisome number, size, and intensity per yeast cell in *Saccharomyces cerevisiae*	Fluorescence microscopy	–	Yes
PeroxiDynA [98]	Median filtering, background subtraction, Otsu thresholding, and watershed separation	Quantification of peroxisome morphology (size, circularity, area, and perimeter) and spatial distribution in mammalian cells under different genetic conditions	Confocal microscopy	–	No

**Fig. 5.**
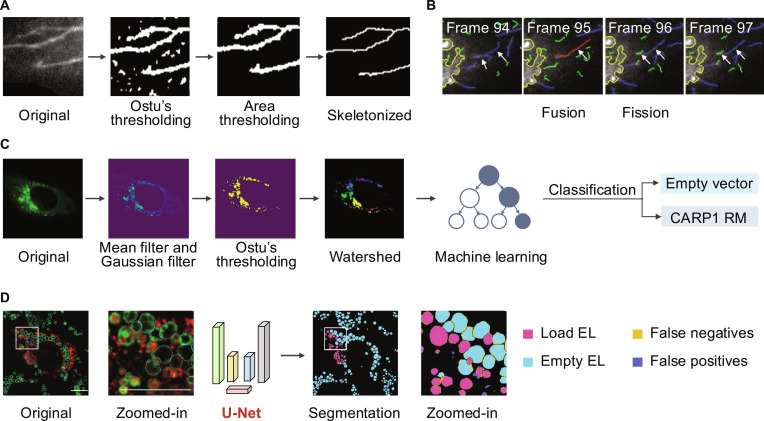
GA and lysosome image segmentation and analysis in living cells. (A) The workflow of 2D-GolgiTrack begins with the original GA image, followed by Otsu’s thresholding to binarize the image. Area-based thresholding is applied to isolate relevant regions, and the resulting structures are skeletonized to extract the features of the Golgi network. (B) The GA tracking results of 2D-GolgiTrack, including fusion and fission events [90]. (C) Sansaria et al. developed a method for processing GA images using mean or Gaussian filtering followed by Otsu’s thresholding for initial segmentation. The watershed algorithm refines segment boundaries, and features extracted from these regions are analyzed with a machine learning model to classify Golgi dispersal patterns into distinct categories [91]. (D) LysoQuant employs a U-Net deep learning model to segment lysosome structures, distinguishing loaded endolysosomes (EL) from empty EL categories [92]. Reprinted from Ref. [90] with permission from Springer Nature; from Ref. [91] with permission from Elsevier; and from Ref. [92] with permission from the American Society for Cell Biology.

Recent advancements in GA image segmentation have focused on tools for analyzing its structural dynamics and role in membrane trafficking. In 2022, Yaothak et al. [90] introduced 2D-GolgiTrack, a semiautomated system designed to quantify morphological changes and dynamic behaviors of the GA and its associated tubules. This tool combines Otsu’s thresholding with adaptive local normalization to segment Golgi components, skeletonize tubules, and track fission and fusion events with over 95% accuracy (Fig. 5A and B). Building on these efforts, Sansaria et al. in 2024 [91] developed a machine learning-based framework for classifying dispersed GA images under stress conditions. This approach employs Otsu’s thresholding and watershed segmentation to extract features such as area, perimeter, and eccentricity to quantifying GA fragmentation. By integrating logistic regression and random forest classifiers, the framework achieved high accuracy in distinguishing GA dispersal patterns, demonstrating its utility for studying stress-induced morphological changes (Fig. 5C).

For lysosomal segmentation, Morone et al. [92] in 2020 introduced LysoQuant, a deep learning-based framework designed for analyzing lysosomal degradation pathways. Built on a U-Net architecture, LysoQuant enables accurate segmentation and classification of endolysosomal compartments from fluorescence microscopy images. The tool quantifies lysosomal number, size, and shape while distinguishing between empty and cargo-loaded lysosomes (Fig. 5D). LysoQuant has proven instrumental in studying ER-phagy and lysosomal clearance of misfolded proteins, providing quantitative insights into catabolic pathways and their regulation. Its integration into an ImageJ plugin streamlines the analysis process, offering researchers a high-throughput solution for investigating lysosomal dynamics and their role in maintaining cellular homeostasis.

LDs appear as bright punctate structures that often cluster or fuse, which complicates instance segmentation in live-cell imaging. Thresholding is effective for isolated LDs, whereas deep learning-based methods perform better for clustered or heterogeneous populations. In 2014, Dejgaard and Presley [93] developed a segmentation algorithm combining thresholding and watershed refinement, which effectively separated clustered LDs and enabled accurate single-cell quantification in fluorescence images. In 2021, Li et al. [94] introduced a hybrid approach combining an improved watershed algorithm with transfer learning-based CNNs for LD recognition, effectively resolving droplet adhesion and enabling high-accuracy classification in pathological images. Sheneman et al. [95] combined label-free quantitative phase imaging with supervised machine learning for single-cell-level LD identification. Results show that U-Net-based CNNs outperformed traditional methods in prediction accuracy. In 2024, Oh et al. [96] proposed a deep learning-based segmentation and pixel-counting pipeline to estimate lipid content in real time. Using U-Net and HRNet with data augmentation and voting fusion, the model achieved 98% cell and 92% LD detection accuracy.

Peroxisomes are small, dynamic organelles involved in lipid metabolism and ROS detoxification, but their low brightness and size make segmentation difficult under low SNR. In 2024, Neal et al. introduced perox-per-cell, an open-source tool for automated segmentation and quantification of peroxisomes in yeast microscopic Z-stack images [97]. The workflow identifies cells and punctate peroxisomes, assigns each to its corresponding cell, and outputs per-cell metrics such as counts, areas, and fluorescence intensities, which closely matched manual measurements and demonstrated suitability for high-throughput analysis. More recently, Ferreira et al. [98] developed PeroxiDynA, a semiautomated ImageJ macro that applies Otsu thresholding and watershed segmentation to analyze peroxisome morphology and spatial distribution in mammalian cells, achieving accurate delineation of clustered organelles from confocal images. These approaches facilitate quantitative evaluation of peroxisome dynamics, improving segmentation reliability and accessibility for nonexpert users.

Research on such segmentation tasks remains relatively less developed compared with mitochondria, ER, and nuclei. Existing approaches for GA segmentation primarily employ thresholding-based techniques, which have proven to be effective in various settings. Complementary techniques based on deep learning are also being explored, with the potential to address complex morphologies and dynamic behaviors. Integrated analysis pipelines that simultaneously address segmentation, classification, and tracking may provide insights into GA dynamics, lysosomal autophagy, LD turnover, and peroxisomal remodeling to underlying metabolic and degradative processes.

### Multi-organelle image segmentation

The coordination among multi-organelles ensures the harmonization of cellular functions, preserving vitality and stability within the cell. These organelles are interconnected through physical and functional interactions, enabling processes such as protein modification, energy production, intracellular transport, and degradation [99]. While single-organelle segmentation provides detailed insights into specific structures, multi-organelle segmentation offers a holistic perspective by simultaneously analyzing the spatial, functional, and dynamic relationships between organelles. This approach minimizes biases introduced by separate analyses and provides a comprehensive framework for studying organelle interactions [57]. Notably, optimal algorithms are often organelle-specific because different organelles exhibit distinct structural and dynamic characteristics [100]. The ER has a highly interconnected network architecture that benefits from topology-preserving methods. Mitochondria undergo continuous fusion–fission cycles and morphological remodeling, which require algorithms robust to shape variability and branching dynamics [101]. Lysosomes, by contrast, are small and nearly spherical, demanding precise instance separation. Accurately segmenting organelles with diverse morphologies and dynamics within a single model remains a major challenge. Additionally, robust segmentation algorithms must maintain accuracy across diverse imaging datasets and experimental settings. To address these challenges, it is essential to develop generalized and efficient segmentation pipelines. These pipelines should integrate quantification and analysis capabilities, enabling comprehensive investigations of the complex interactions between multiple organelles (Table 7).

**Table 7.** Summary of representative algorithms and techniques for multi-organelle image segmentation and analysis

Algorithm	Technique	Applications	Modality	Performance	3D capability
Huang et al. [100]	Multiresolution encoder and hierarchical fusion loss	Segmentation of ER and mitochondrial networks COS-7 cells. Applied to study dynamic morphological and topological properties of these networks.	Fluorescence microscopy	ER: IoU = 0.77; Mitochondria: IoU = 81.21	No
OrgaMeas [102]	U-Net and pix2pixHD	Segmentation and quantitative analysis of mitochondria, lysosomes, lipid droplets, and nuclei in HeLa cells	Differential interference contrast microscopy	Dice = 0.96–0.97	No
Nellie [103]	Multiscale structural contrast enhancement, motion capture markers, and sub-voxel flow	Analysis of diverse intracellular structures across multiple cell types. Use cases include feature-based differentiation of organelles in single-channel images.	Single objective light sheet, spinning-disk confocal, and wide-field microscopy	Segmentation: On simulated datasets, IoU > 0.75 Organelle unmixing: AUC > 0.80	Yes

**Fig. 6.**
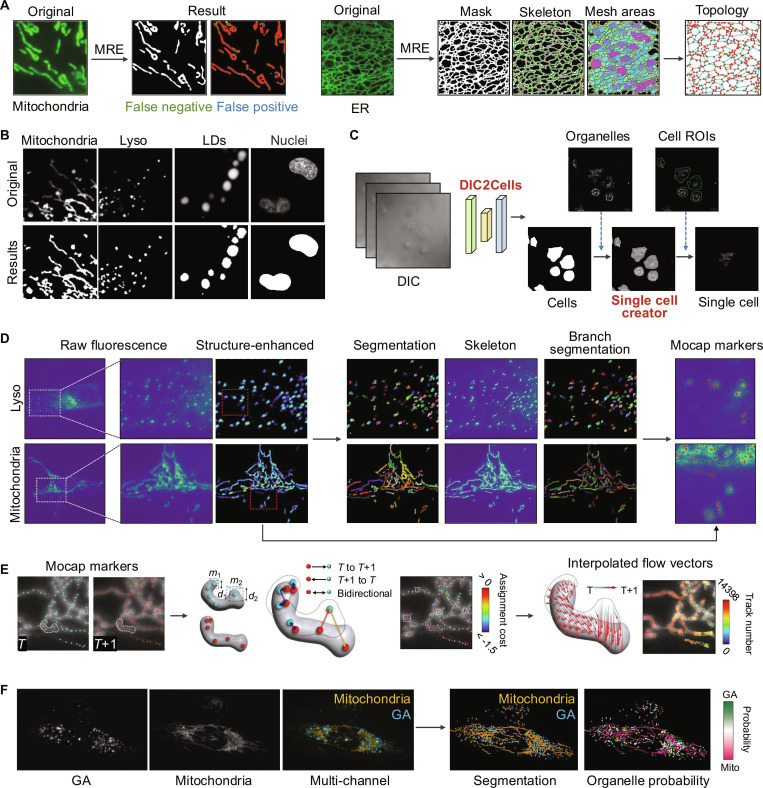
Multi-organelle image segmentation and analysis in living cells. (A) MRE segments the original fluorescence image of mitochondria and ER using a multiresolution encoder (MRE) algorithm. The topology of the ER network is analyzed, displaying critical features such as 3-way junctions and connectivity graphs for understanding ER structural organization [100]. (B) OrgaMeas is used to segment mitochondria, lysosomes, lipid droplets, and nuclei from fluorescence images, allowing for detailed multi-organelle quantification. (C) OrgaMeas’s expanding pipeline DIC2Cells, enabling segmentation of organelles at the single-cell level, integrating differential interference contrast (DIC) images [102]. (D) Nellie enhances structural contrast in organelles, from spherical lysosomes to complex mitochondrial networks, using multiscale filtering algorithms. Instance segmentation separates connected components, while skeletonization enables branch-based sub-compartment analysis, preserving spherical structures and introducing hierarchical substructures for networks. Motion capture (mocap) markers are detected via multiscale peak detection within segmentation masks. (E) Nellie analyzes mitochondria and their mocap markers across consecutive time points (*T* and *T*+1). Feature search bounding boxes (*m*_1_, *m*_2_) with radii (*d*_1_, *d*_2_) are defined at *T*, and markers are linked between frames based on assignment cost. Interpolated flow vectors, represented by arrows, illustrate mitochondrial motion between *T* and *T*+1. (F) Nellie combines raw GA and mitochondrial intensity images into a multichannel fluorescence signal. A trained random forest classifier generates binary organelle predictions with region-specific probabilities, enabling quantitative analysis of GA and mitochondrial features [103]. Reprinted from Ref. [100] with permission from Oxford University Press; from Ref. [102] with permission from Elsevier; and from Ref. [103] with permission from Springer Nature.

In 2024, Huang et al. [100] proposed the Multi-Resolution Encoder (MRE), a deep learning framework designed to enhance segmentation of ER and mitochondrial networks by leveraging low-level features and topological self-similarity. By introducing hierarchical fusion loss and multiscale random cropping, MRE improves segmentation accuracy for complex organelle networks, achieving superior performance on ER and mitochondrial datasets. Additionally, their method facilitates topological and morphological analysis, offering novel insights into organelle dynamics and functions (Fig. 6A). This approach represents a specialized integration designed for concurrent analysis of the ER and mitochondria, primarily optimized for network-like structures and thus requiring further adaptation to generalize to other organelle morphologies. In parallel, Baba et al. [102] introduced OrgaMeas, a comprehensive pipeline that integrates segmentation, ROI setting, and quantification for diverse organelles, including mitochondria, lysosomes, LDs, and nuclei (Fig. 6B). The pipeline employs OrgaSegNet, a U-Net-based model that integrates MitoSegNet and NucSegNet for precise segmentation. Subsequently, DIC2Cells, a pix2pixHD-based conditional adversarial network for automated single-cell ROI generation, enable high-throughput and single-cell-level analysis (Fig. 6C). OrgaMeas has demonstrated its utility in quantifying morphological changes in response to various experimental conditions, such as mitochondrial fragmentation and lysosomal activation.

While both studies address challenges in multi-organelle segmentation, they share common limitations. MRE focuses specifically on ER and mitochondrial networks, limiting its generalizability to other organelles. OrgaMeas, although versatile, relies heavily on pretrained models that are tailored to specific datasets and imaging conditions, making it less adaptable when applied to organelles with different structural or signal characteristics. Its generalizability further depends on the availability of well-annotated data, and extending it to new organelles requires the development of additional specialized modules. These limitations underscore the need for more comprehensive and generalized segmentation pipelines that can adapt to diverse organelles and imaging modalities while ensuring high accuracy, robustness, and efficiency.

The primary challenge in multi-organelle segmentation is the morphological variability among organelles. This heterogeneity limits the effectiveness of deep learning models, which typically require large and homogeneous training sets. In contrast, thresholding-based methods, owing to their efficiency and inherent adaptability, remain a viable option for addressing this task. In 2025, Lefebvre et al. [103] developed Nellie, an automated pipeline integrating multiscale preprocessing, hierarchical segmentation, and motion tracking for robust analysis across diverse organelles and imaging modalities. Nellie employs the “Minotri” adaptive thresholding method to generate semantic masks and skeletonization to decompose complex organelle structures into sub-compartments (Fig. 6D). For motion tracking, the pipeline incorporates a motion capture marker (mocap)-based linking system, using local pattern matching and multiscale peak detection to achieve sub-voxel accuracy in temporal interpolation and motion tracking (Fig. 6E). Additionally, Nellie uses graph representations of segmented organelle networks to separate signals from multiple organelles, such as the GA and mitochondria, within single-channel fluorescence images (Fig. 6F). The pipeline’s hierarchical segmentation and graph autoencoder were applied to investigate mitochondrial network dynamics under ionomycin treatment, validating Nellie’s practical utility for multi-organelle research.

Multi-organelle image segmentation represents a frontier in cellular imaging analysis, offering the potential to uncover intricate inter-organelle relationships. Future efforts could focus on developing multitask frameworks capable of segmenting multiple organelles simultaneously while preserving their spatial and functional context. Incorporating graph-based representations may enable modeling of complex networks formed by interacting organelles. Additionally, leveraging transfer learning techniques could accelerate model adaptation to diverse imaging modalities and experimental conditions. Real-time segmentation paired with dynamic modeling approaches will further support the exploration of temporal interactions among organelles, paving the way for comprehensive studies on subcellular coordination.

## Discussion and Outlook

The integration of live-cell imaging with segmentation technology has markedly enhanced the granularity and fidelity of subcellular process studies. Unlike fixed-cell approaches, live-cell imaging introduces a temporal dimension, capturing of dynamic events such as organelle trafficking and fusion–fission cycles [8–12]. When paired with image segmentation algorithms, it enables quantitative tracking subtle morphological changes, phenotypic shifts, and precise spatiotemporal localization of biological targets [46].

Despite substantial advancements, image segmentation still faces challenges that limit its universal applicability. Robustness and generalizability remain critical issues, particularly across diverse imaging modalities [104]. Additionally, subcellular structures often exhibit intricate architectures that vary notably in size, shape, and fluorescence intensity depending on experimental conditions, labeling strategies, and the optical properties of the imaging system [105,106]. Meanwhile, the dynamic nature and morphological complexity of organelles and highly overlapping or fractal-like structures present additional hurdles. Addressing these challenges requires more than incremental algorithmic refinements to improve generalizability and domain adaptation.

Generalizability can be strengthened by training models to capture biological structures and the specific characteristics of diverse imaging systems, including variation in resolution, contrast, and optical artifacts [107]. Achieving this demands models that can effectively transfer learned features across imaging modalities. Domain adaptation strategies, such as adversarial training, fine-tuning, and physics-informed augmentation, can bridge the gap between training and target new domains by aligning feature distributions across datasets and conditions [108]. These strategies improve robustness across diverse experimental setups and reduce sensitivity to system-specific biases. Furthermore, the absence of standardized evaluation metrics has hindered meaningful comparisons between segmentation methods. Establishing comprehensive, organelle-stratified benchmarking datasets with unified evaluation protocols will enable consistent and objective assessment of segmentation performance across organelles and imaging modalities.

3D imaging data add substantial complexity by capturing the full spatial context of subcellular structures while imposing heavy computational demands due to massive voxel counts. Anisotropic resolution and uneven signal attenuation further complicate segmentation, and the overlapping nature of 3D structures makes boundary identification more challenging than in 2D projections [109]. Additionally, annotating 3D data is laborious, and the scarcity of high-quality training sets poses a severe bottleneck for deep learning-driven solutions [110]. Addressing this complexity necessitates reducing reliance on large annotated training sets by adopting self-supervised or transfer learning approaches, and implementing online or incremental learning frameworks that enable models to dynamically adapt to fluctuating imaging conditions and newly acquired data. Further integration of cloud-based computing and more efficient graphics processing unit (GPU) acceleration can expedite the iterative cycles of model training, validation, and refinement, ultimately advancing the scalability and accuracy of 3D segmentation in complex biological systems. However, leveraging these computational resources also requires addressing fundamental imaging artifacts, particularly the anisotropic resolution common in 3D microscopy. This anisotropy can distort structural morphology and mislead segmentation algorithms [111]. To address these distortions, self-supervised learning presents an effective strategy by pretraining models on large, unlabeled 3D datasets to capture intrinsic, orientation-invariant morphological features of organelles [112]. This approach reduces model dependence on anisotropic signal distributions and improves robustness across diverse imaging conditions.

Although deep learning has demonstrated impressive segmentation accuracy, it is critically dependent on large, well-annotated datasets. The generation of such datasets is both labor- and resource-intensive, prompting the exploration of alternative learning strategies. This dependency has spurred interest in alternative strategies that reduce manual annotation. Virtual labeling, which uses computational models and empirical measurements to generate training data, shows promise by refining internal representations and enhancing generalization to complex imaging scenarios [113]. In parallel, physics-informed models incorporate theoretical optical principles and biophysical constraints into the segmentation process, guiding model training toward physically plausible outcomes, even with limited high-quality labels [114]. Furthermore, few-shot learning addresses the issue of limited training data, allowing models to adapt efficiently to new organelle classes, imaging modalities, or perturbation conditions using minimal examples [115]. Together, these emerging paradigms aim to reduce the burden of manual labeling while fostering algorithms that are more flexible, data-efficient, and capable of analyzing the intricate complexities of subcellular environments.

Notably, these approaches rely on specific prior assumptions, and their effectiveness may be limited by the domain gap between simulated or virtually labeled data and real experimental images [116]. To complement simulation-based methods, automated annotation strategies have emerged as a practical alternative that programmatically generate large-scale pseudo-labels. Common approaches include pseudo-labeling, weak supervision, bootstrapping, and active learning, all of which aim to shift expert effort from exhaustive labeling to verification and correction. In practice, reliable deployment combines pseudo-labels with confidence filtering, noise-robust losses, curated fine-tuning, or domain adaptation to mitigate label noise and improve generalization [117].

An exciting development in image analysis is the application of large-scale generalist models, such as the SAM, and their derivatives to biomedical segmentation pipelines [118–120]. Originally designed for natural imagery, these architectures generalize well across diverse imaging conditions, structural complexities, and resolution scales. By incorporating domain-specific priors, fine-tuning routines, or specialized training schemes, SAM can be adapted to sensitive biological contexts with minimal additional annotation [121]. For instance, segment anything for microscopy enhances segmentation performance across multiple imaging modalities by fine-tuning generalist models on a diverse microscopy dataset [122]. This paradigm shift points toward a future where a pretrained, universally applicable segmentation backbone underpins a wide array of microscopy workflows, accelerating development cycles and enhancing reproducibility. As the ecosystem of SAM-based derivatives continues to mature, researchers stand to benefit from streamlined data processing, standardized evaluation protocols, and accelerated innovation in imaging-based investigations.

Advances in segmentation have the potential to drive a quantitative paradigm shift in cell biology. These methods transform image analysis from descriptive observation into statistically rigorous measurement of subcellular morphology and dynamics [123]. By enabling 3D, time-resolved, and multi-organelle quantification, these techniques also open practical opportunities for AI-assisted pathology and imaging-based, high-throughput phenotypic screening in drug discovery. In pathology, robust segmentation transforms visual assessments into quantitative descriptors, including morphology, density, and spatial patterns of immune infiltration. In drug discovery, segmentation supports high-content phenotypic screening by automating the extraction of organelle-level features [124]. This process enables the generation of dose–response curves and the clustering of morphological signatures for mechanism-of-action inference. Moreover, ethical considerations deserve explicit attention. Biomedical imaging datasets may contain sampling and labeling biases that can introduce systematic errors in downstream analyses. To ensure reliable deployment, models should be validated across diverse biological samples and imaging conditions [125]. Evaluation should also include uncertainty calibration, failure-case analysis, and transparent documentation of data provenance.

In summary, the integration of AI and advanced microscope imaging techniques has recently achieved remarkable progress in organelle segmentation. AI has demonstrated its crucial role in improving segmentation accuracy and facilitating the analysis of increasingly intricate organelle images. Furthermore, these computational advancements offer considerable potential for minimizing time and resource consumption, thereby broadening the application of image segmentation in life sciences.
